# A deep learning framework for histopathological analysis of pixel-level extracellular matrix variation in standard H&E-stained images

**DOI:** 10.1038/s41598-026-56138-9

**Published:** 2026-06-22

**Authors:** Merlijn van Breugel, Esmée de Jong, Henk J. Buikema, Ilya Petoukhov, Martijn C. Nawijn, Janette K. Burgess, Wim Timens

**Affiliations:** 1 Department of Pediatric Pulmonology and Pediatric Allergology, University of Groningen, University Medical Center Groningen, Beatrix Children’s Hospital, Groningen, the Netherlands; 2 Ditto Care, Rotterdam, the Netherlands; 3 Rewire, Amsterdam, the Netherlands; 4 Department of Pathology & Medical Biology, University of Groningen, University Medical Center Groningen, Groningen, the Netherlands; 5 A Beta World, Utrecht, the Netherlands; 6 Groningen Research Institute for Asthma and COPD, University of Groningen, University Medical Center Groningen, Groningen, the Netherlands; 7 Department Pathology and Medical Biology, hpc EA10 University Medical Center Groningen, Hanzeplein 1, Groningen, 9713 GZ The Netherlands

**Keywords:** Digital pathology, Deep learning, Extracellular matrix, Whole-slide image, Chronic obstructive pulmonary disease, Transfer learning, Computational biology and bioinformatics, Diseases, Engineering, Mathematics and computing, Medical research

## Abstract

Artificial intelligence-driven image analysis has enabled significant advances in digital pathology. However, most approaches have focused on cell or organ structures. This manuscript presents a reproducible deep learning methodology for pixel-level analysis of amorphic patterns in haematoxylin and eosin-stained whole-slide histological images. This study analysed the pixel patterns in the extracellular matrix (ECM) part of connective tissue to identify differences in airway wall ECM compartments and their heterogeneity, which are microscopically similar and difficult to discern with the human eye. Through a targeted preprocessing pipeline, the deep learning model is guided to emphasise learning from pixel-level patterns in non-cellular tissue components while reducing the influence of cellular structures and artefacts. Combined with transfer learning, the model accurately distinguishes the characteristics of the airway submucosa and adventitia, achieving a test area under the curve of 0.84. Using visualisation techniques and statistical analysis, we demonstrate that random pixel imputation successfully reduces the effects of cellular structures on model learning. The framework is applied in a proof-of-principle study of lung tissue from patients with chronic obstructive pulmonary disease, illustrating how this quantitative approach can study population heterogeneity and inform novel research directions. Ultimately, this study provides an innovative and adaptable framework that unlocks the analytical potential of often-overlooked amorphic components in AI-empowered histopathology.

**Supplementary Information:**

The online version contains supplementary material available at https://doi.org/10.1038/s41598-026-56138-9.

## Introduction

Artificial intelligence (AI) is driving innovations in the field of medical image analysis. By leveraging the steep increase in computing power, the availability of high-resolution image data, and deep learning algorithms^1,2^, AI-driven image analysis has enabled advances across many medical domains in recent years. Whereas initial advances were mainly in radiology image assessment, these advancements are now more generally applicable. AI has also proven to be a valuable new tool in digital microscopy image research. Digital pathology is progressing rapidly due to the availability of high-resolution whole slide image (WSI) scanners and increased storage capacity^3,4^. These developments have enabled and promoted successes in image recognition and analysis applications^5–9^, with algorithms developed to accurately detect cancer tumour regions in whole slide images^10–12^, recognise various tissues^13,14^ and even predict tumour mutations from standard haematoxylin and eosin (H&E) microscopy images^15^.

The vast majority of these studies have focused on supervised learning tasks^16^, where the image recognition algorithm is trained to classify, diagnose or detect known patterns, such as the presence of a tumour in a lung. In addition to their use in the diagnostic pathology field, these approaches also offer new insights into the mechanisms of disease^17^. Until now, most of these approaches have learned from cellular shapes^18^. Image recognition research specifically focused on the pixel-level analysis of amorphic components, such as the extracellular matrix (ECM), is an area that has yet to be explored. The ECM defines the structure of connective tissue and plays an important role in organ function by providing structural integrity, defining mechanical properties, and regulating communication to and between cells^19,20^. As such, changes in the composition and mechanical or biophysical features of the ECM are among the driving forces contributing to human disease pathogenesis^21^. Hence, studying the different features of the ECM contributes to a better understanding of the complex biological dynamics of diseases^22^. In turn, this may lead to new insights and use in diagnostic pathology.

Despite its important function within the human body, visual observation of changes and variations in the ECM in standard and broadly used haematoxylin and eosin (H&E) histochemical staining is notably difficult for two main reasons. First, structural differences in the ECM are challenging to detect because of the limited distinguishable shapes and (for the human eye) narrow range of H&E staining characteristics, such as variations in colour or intensity. Second, the changes induced by many diseases in the ECM are characterised by a large degree of heterogeneity in the contributing ECM proteins^23^, which is not readily apparent from the stain in the diagnostic H&E-stained images. To date, it is largely unknown whether image recognition algorithms can enhance the analysis of complex ECM structures, constituting the connective tissue that, in standard histopathology, H&E stains are difficult, if not impossible, to discern with the human eye. This study hypothesises that deep learning approaches can be used in standard tissue H&E histopathology to identify differences in ECM patterns associated with disease and disease heterogeneity.

**Fig. 1:**
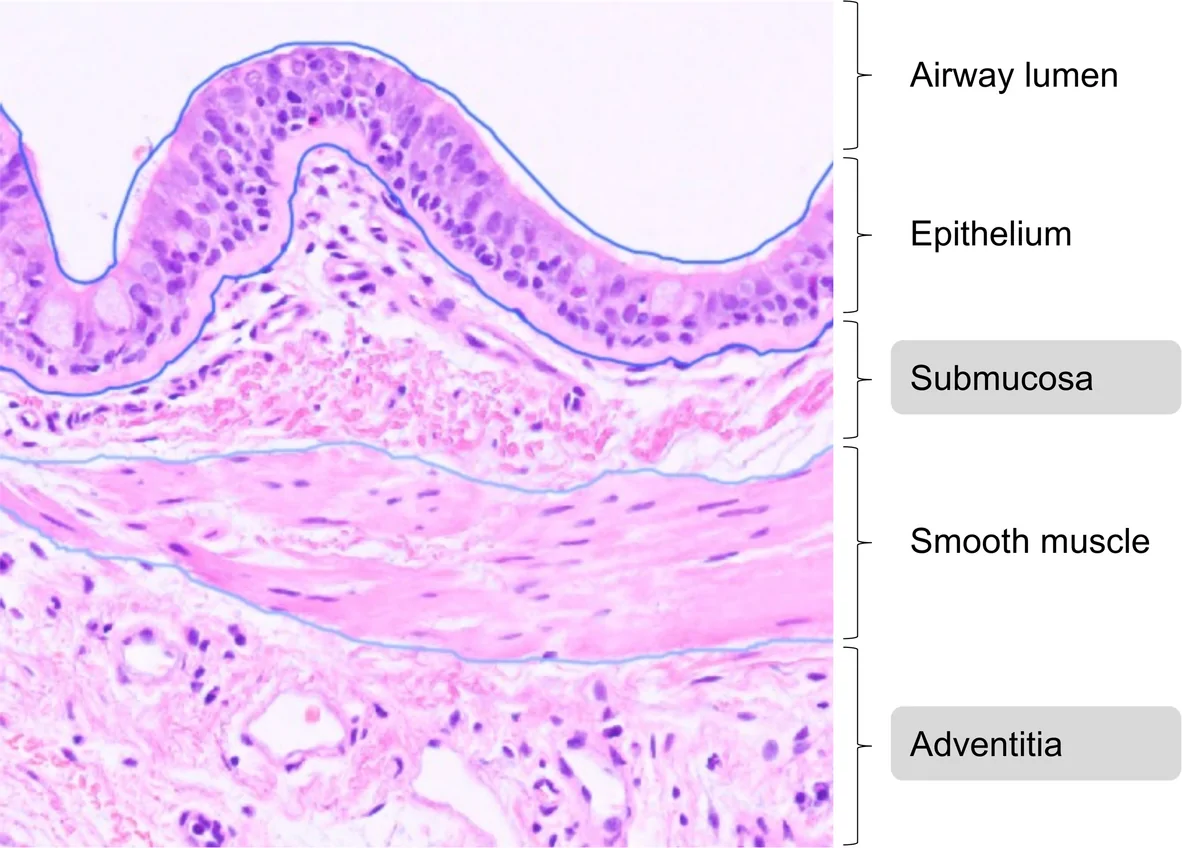
Visualisation of the submucosa and adventitia lung compartments. The lung compartments are separated by the airway wall and smooth muscle, where the submucosa ranges between the epithelium with the basement membrane and the smooth muscle, and the adventitia starts from the smooth muscle downwards to the bordering alveoli.

To test this hypothesis, this study evaluated the feasibility of an image analysis approach to identify patterns in H&E-stained lung sections, such as those present in amorphic ECM structures in the airway wall in chronic obstructive pulmonary disease (COPD), a respiratory disease with ECM changes in the small airway walls^24,25^. In COPD patients, these changes are characterised by disrupted tissue repair mechanisms and excessive deposition of structural proteins, among other effects, leading to airway wall remodelling and compromised lung function. A deep learning model is developed to classify airway wall components (Fig. 1) by analysing numerous image patches from both COPD and control subjects. Deep learning is a subdiscipline of machine learning that leverages neural networks, which are powerful models that are used predominantly to analyse unstructured data such as images. For the image data, WSIs of airway walls derived from H&E-stained lung tissue were used. The prediction outcomes provided the basis for a proof-of-principle to quantify and compare ECM patterns at the pixel level within and across tissue compartments and patient groups, demonstrating how this study’s methodology can be used to explore new research questions.

## Methods

**Fig. 2:**
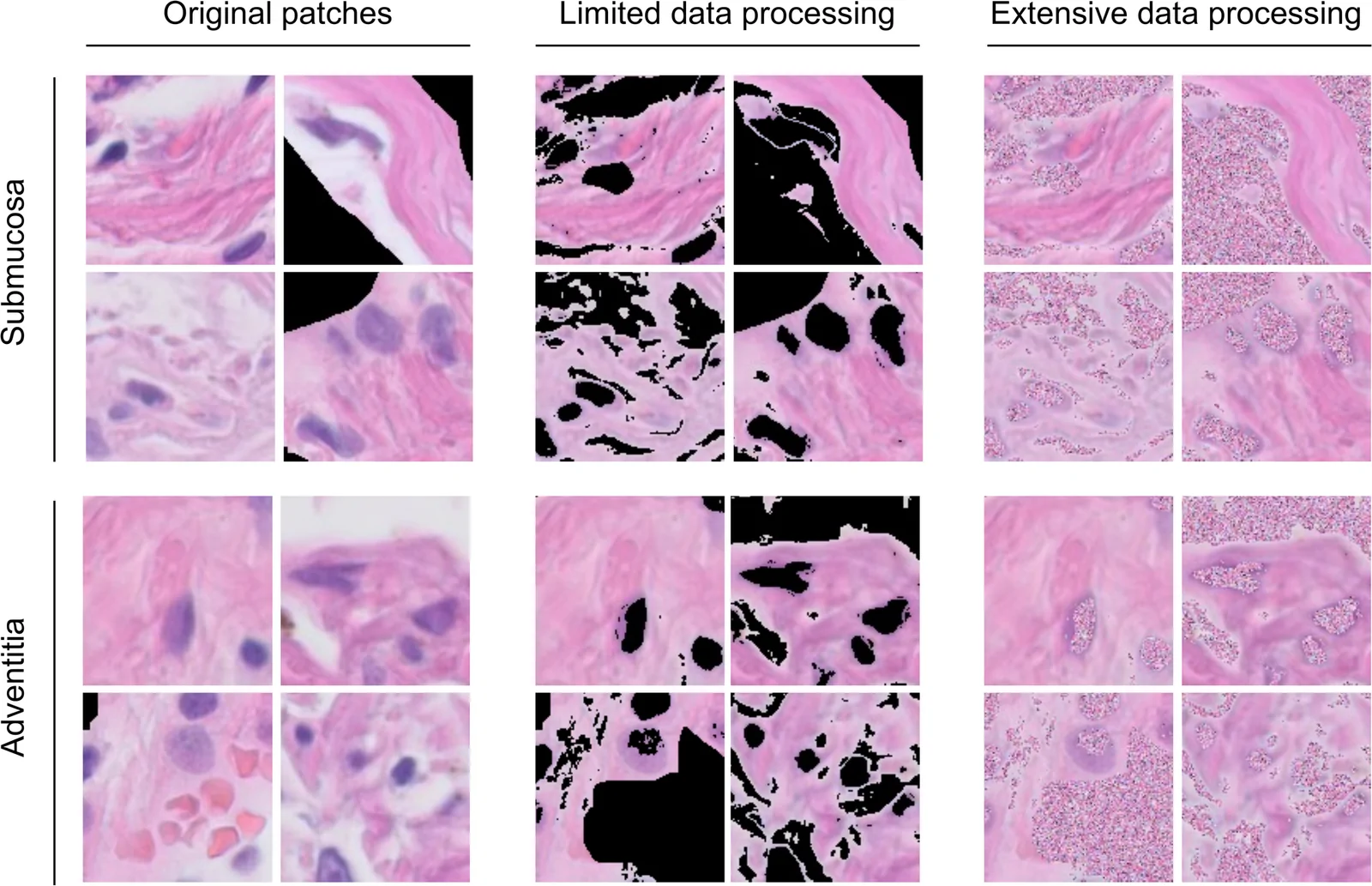
Procedure to perform automatic pixel-level comparison of different tissue sections. The procedure consists of three steps: (**A**) data preprocessing, (**B**) model training, and (**C**) model interpretation. Architecture in panel (B) was partly generated via NN-SVG^63^. For reference, the actual size of the first image in panel A is 2.0 × 1.9 mm in length and width, respectively. .

This study’s methodology for image analysis uses a three-step approach to perform automatic pixel-level comparison of H&E-stained tissue sections (Figs. 1 and 2): data acquisition and preprocessing (Fig. 2a, Supplementary Fig. 1), model training (Fig. 2b) and model interpretation (Fig. 2c). Each of these steps is discussed in sequential order below, with further details included in the Supplementary Methods.

### Dataset

A total of 162 WSIs of H&E-stained cross-sectional airway sections from 75 patients from the archive of the Department of Pathology and Medical Biology of the University Medical Center Groningen were coded (pseudonymised). Patients underwent either surgical lung resection for COPD (lung transplants) or for lung cancer, the latter with either COPD GOLD stage 1 or 2 or normal lung function. Patient characteristics, including smoking history and lung function, are provided in Supplementary Table 1.

Glass slides were scanned in a Philips Ultra Fast Scanner 1.6 (Philips, Eindhoven, the Netherlands) and captured at 40x magnification using a single-focus layer without Z-stacking. Tissue detection with focus points was applied automatically to obtain the optimal image. The digitised slides were stored on a centralised server. The WSIs were converted to BigTIFFs with a resolution of 0.25 micrometres per pixel (mpp). All WSIs containing noncartilaginous airways (bronchioles) and far distant from cancer involvement were included in a consecutive series of cases without further selection.

### Image annotation and preprocessing

#### Annotation (Fig. 2, steps 1.1–1.5)

For any downstream analysis, obtaining qualitative annotations of the WSIs is a critical step^7^. To annotate the region of interest, i.e., the submucosa and adventitia (explained in Fig. 1), first, manual annotations of noncartilaginous airway walls are performed in Qupath (version 0.2.0)^26^. The annotation included the respiratory epithelium and airway smooth muscle, marking the boundaries of the two key lung compartments of interest (Fig. 1). All the regions included where the submucosa and adventitia could be recognised by their separation by airway smooth muscle. For each region, three PNG files were exported using the original resolution of 0.25 mpp: the selected tissue section and two corresponding binary masks (black and white images) of the annotated epithelium and smooth muscle. Annotation was executed primarily by a digital pathology technician and validated by a pathologist, who also confirmed equivalent respiratory tree depth in the samples. Initial data preprocessing commenced on 1 February 2021.

#### Segmentation (Fig. 2, step 2)

All further data acquisition and preprocessing steps are built as an automated preprocessing pipeline in Python (version 3.11). To separate the submucosa from the adventitia in the exported tissue section images from QuPath, binary masks are applied to the mucosa and smooth muscle in the corresponding tissue image. For each image, this resulted in two compartment-distinctive images, where either the submucosa or the adventitia was represented. These images were saved in compartment-specific folders for data patching to equalise the image size and resolution.

#### Patching (Fig. 2, step 3)

After segmentation, a sliding window method is applied to split the compartment-distinctive images into equally sized patches of 120 × 120 pixels^27^. This procedure slides with steps equal to the image size, thereby ensuring that there are no overlapping patches. Since an image recognition model requires a predefined input dimension, equalising the patch size ensured that an identical resolution was maintained across all the model input data. The relatively small patch size was used to account for the relatively limited surface area of the submucosa and to extract full tissue patches from both compartments. Because of differences in thickness between the two tissue layers of interest, more adventitia patches were extracted compared to the submucosa. All patches were consecutively coded and completely anonymised for use in cloud systems.

#### Cleaning (Fig. 2, steps 4–5)

Pilot analyses revealed that shapes, irrelevant for the aim of this study, such as the epithelium and clusters of red blood cells (RBCs), could drive compartment classification. To enforce pixel-level analysis of the ECM during model training and avoid focusing on cellular and non-ECM structures, the present work combines three data cleaning approaches in sequential order.

1. Masking of cellular structures and artefacts.
2. Remove noninformative patches with many cellular and non-ECM structures.
3. Perform pixel randomisation to replace the masked areas in the remaining patches.

In the first data cleaning step, this method replaced cellular structures, including RBCs, lymphocytes and the epithelium, as well as artefacts, such as white backgrounds and carbon particles, with black pixels. RGB ranges are used to detect the purple-stained cellular structures that need to be masked (Supplementary Table 6). Since RBCs are not very well distinguishable by colour from the ECM, QuPath’s built-in cell detection algorithm is employed to produce masks of RBCs. Furthermore, random pixel replacement is performed to deconstruct shape-related patterns induced by the masks and further force the model to learn from the unstructured ECM. All black pixels were randomly replaced by pixels sampled from the non-black (tissue) pixels within the same patch. This per-patch imputation ensures that no colour distribution information is shared between patches or across data partitions.

This methodological approach subsequently determines a threshold for the ratio of irrelevant cellular and non-ECM structures in the patches and evaluates how the two noise cleaning approaches affect the quantity and quality of the data. For each threshold in the set (20%, 30%, …, 80%), two datasets were created for both approaches: a dataset containing black-imputed noise by skipping the random pixel imputation step and a dataset containing randomly imputed noise following the developed cleaning approach. Patches containing fewer than the given threshold of tissue pixels after step 1 were removed from the datasets. Patches with percentages less than 20% were considered too uninformative, whereas setting a strict threshold above 80% removed almost all patches. Supplementary Fig. 2 depicts the total number of patches of 120 × 120 pixels available for model training and evaluation per threshold, with the proportions for the adventitia and submucosa, respectively. The distributions of patches according to patient characteristics can be found in Supplementary Table 2. Prior to model training, the data were split into training, validation and test sets at the patient level, ensuring that all WSIs from a given patient reside in the same partition. The respective proportions equalled 60%, 20% and 20% of patients, stratified by COPD group (control, GOLD III, GOLD IV) so that each partition preserves the severity distribution of the full cohort. A comparison of patch-level, WSI-level, and patient-level splitting is presented in Tables 1, 2 to justify this choice; all other results use patient-level partitioning.

**Table 1:** Model performances on the training, validation and test sets for different transfer learning settings.

Transfer learning type	# Trainable layers (parameters)	Train AUC	Validation AUC	Test AUC
Classification layer	3 (1,574,913)	0.7668	0.7511	0.7188
Inception-ResNet layer block C	151 (24,016,289)	0.8481	0.7961	0.7761
Inception-ResNet block B	494 (50,810,849)	0.9363	0.8316	0.8142
Inception-ResNet block A	722 (55,329,393)	0.9404	0.8478	0.8249
Input layer	781 (55,851,105)	0.9369	0.8472	0.8215

The Inception-ResNet model architecture is primarily constructed of three sequential sets of layers, which are termed blocks A–C^28^. This study’s transfer learning procedure included an increasing number of retrained layers and parameters. When block A is trained, which is the first block after the input layer, all the following blocks are required to be retrained, as is also represented by the incremental number of trainable layers. The hyperparameters were not individually optimised for these models; default values, as stated in Sect. 2.3.2, were used. All models were trained on 26,152 patches from 43 patients (training set) and evaluated on 8,752 patches from 12 patients (validation) and 9,919 patches from 18 patients (test). Two of the 75 patients in the cohort (each contributing a single WSI) yielded no patches satisfying the 20% tissue-pixel threshold and were therefore excluded from model training, leaving 73 patients across 156 WSIs in the partitioned datasets.

**Table 2:** Model performance of the Inception-ResNet-V2 model for different independent types of model training.

Train-validation-test split	Train AUC	Validation AUC	Test AUC
Independent sets on patch level	0.9128	0.8607	0.8663
Independent sets on WSI level	0.9135	0.8365	0.8355
Independent sets on patient level	0.9369	0.8472	0.8215

The independence type refers to the way in which images are split over the training, validation and test sets. Three options are included: (1) random division of patches over the datasets, (2) random division of WSIs over the datasets, and (3) random division of patients over the datasets. All the results were based on Inception-ResNet-V2 with 781 retrainable layers. All results are based on default hyperparameters (Sect. 2.3.2) to ensure a fair comparison across split types. Stability across differently composed patient-level partitions is reported in Supplementary Table 9.

After this extensive data preprocessing, patches can be classified by the model based on ECM tissue patterns instead of (masked) cellular structures such as the airway epithelium, smooth muscle and red blood cells (RBCs).

### Model

#### Architecture

Based on a comparison of pretrained convolutional neural network models commonly used in medical imaging, the Inception-ResNet-V2 model^28^ is selected with pretrained weights on the ImageNet dataset (Supplementary Fig. 9) as the backbone architecture, which is composed of 781 feature extraction and classification layers. This architecture is built on the Inception-V3^29^ and ResNet networks^30^, which already have proven performance using only limited complexity in many medical imaging tasks^31–33^. By integrating residual blocks of ResNet networks into Inception modules, Inception-ResNet-V2 has already been shown to outperform its precedents^28^, and it has also been used for WSIs^27^.

#### Training

The authors trained the model to classify the submucosa and adventitia in a supervised learning setting. This training process was implemented in PyTorch (version 2.4.1, with TensorFlow version 2.17.0 dependency) with CUDA version 12.2 and Weights & Biases (wandb 0.18.1) for experiment tracking. The models have been trained within a GPU-enabled container of RunPod (Dover, Delaware, US), but the setup can also run on the Google Collaboratory platform (Colab). The patch size was upscaled to 224 × 224 via bilinear interpolation to conform to the accepted input of the Inception-ResNet-V2 network. The batch size and number of training epochs are initially set to 64 and 50, respectively. An early-stage warm-up period of 20 epochs is imposed.

For model training, two adaptations were made to the training set. First, the training set was balanced by randomly oversampling the submucosa patches as a minority class to force the model to learn equally well from both classes. Second, image transformations, including random flips, crops and rotations, and colour adjustments, were randomly applied to each batch during model training to improve performance and prevent overfit, including random flips, crops and rotations, and colour adjustments.

The network was trained using the Adam optimizer^34^ with a starting learning rate of 0.001 and default beta decay values of 0.9 and 0.999. Learning rate reduction on plateau was applied based on the validation loss, with a patience of 10, a reduction factor of 0.1, a minimum delta of 0.0001 and a minimum learning rate of 0.00001.

#### Transfer learning

Convolutional neural networks typically consist of multiple layers with corresponding parameters, which can be broadly split into feature extraction and image classification parts (Fig. 2b). Transfer learning is a common strategy to improve model performance by leveraging an existing architecture that has been pretrained on a large dataset such as ImageNet^7^. This work evaluated two transfer learning variations: off-the-shelf transfer learning and additional layer retraining^35^. Off-the-shelf transfer learning refers to only retraining the classification part of the network, whereas for additional layer retraining, layers within the feature extraction part are also retrained. In particular, the latter approach acts upon the potentially limited transferability of the networks trained on the ImageNet database to medical imaging tasks, which is a major criticism of transfer learning^36^. To evaluate the effects of different transfer learning settings, five different models were trained with an increasing number of layers to be retrained.

In all transfer learning approaches, the final classification layer is reinitialised via ReLU activation^37^, a dropout of 0 and an HE normal weight initialiser. For additional preceding layers to be retrained, the extracted pretrained weights are used for initialisation. Different starting points of additional layer retraining were compared using the start and end of the Inception blocks, such that the coherence within and between consecutive blocks of the network was not obstructed.

#### Evaluation

Similarly, different models were trained to determine the optimal image preprocessing approach. The optimal preprocessing and transfer learning settings were determined using the validation AUC of the ROC curve, using patient-level data partitions. The test set was reserved and used only once, after all model selection was finalised, to report the final performance.

To select the best data preprocessing pipeline, two steps are executed sequentially. The first step focused on the effect of thresholding to balance data quantity and quality in model training. For the generated and randomly imputed datasets with varying thresholds from 20% to 80%, models were trained on patient-level data partitions, and the optimal hyperparameters were selected based on the highest validation AUC. Second, the approach is evaluated to control the effect of the remaining noise in the data. For each type of noise handling, black or randomised artefact, a model was trained and evaluated based on the information that the model extracted from noise relative to the information in tissue, and the prediction development with an incremental amount of noise was compared across the datasets. Using both Grad-CAM^38^ and Integrated Gradients (IGs)^39^, information scores are calculated as the average attribution over all noisy pixels, either black or randomly imputed. While less commonly used than Grad-CAM, IG offers a theoretically well-grounded alternative approach to attribution analysis. In contrast to Grad-CAM’s emphasis on final convolutional layer patterns, IG provides more granular pixel-level attribution by analysing complete gradient paths and incorporating an intrinsic baseline.

For noise prediction development, the model’s prediction was recalculated for an incrementally increasing amount of noise injection, ranging from 0 to 100% in steps of 5. To correct for patch- or pixel-specific effects, a subset of 100 correctly attributed patches per lung compartment was selected, and 50 replications were performed per patch and noise level^38^. The effect of the hierarchical data structure was evaluated via the validation AUC by splitting the training, validation and test sets at the patch, WSI and patient levels.

#### Ablation analysis

To further analyse our approach’s contribution over other methods, we performed various ablation analyses. As a negative control, the full pipeline was repeated with randomly permuted compartment labels to verify that the model’s classification performance depends on genuine tissue signal rather than preprocessing artefacts.

As a classical baseline, grey-level co-occurrence matrix (GLCM)^40^ and local binary pattern (LBP)^41^ texture features were extracted from each preprocessed patch and used to train a linear support vector machine (SVM)^42^ on the same patient-level data split. GLCM features (contrast, energy, homogeneity, correlation) were computed at distances 1 and 3, averaged over four angles. LBP features were computed using uniform encoding (*P* = 8, *R* = 1) and summarised as a 10-bin histogram. The SVM regularisation parameter was selected from {0.01, 0.1, 1, 10, 100} on the validation set.

To isolate the contribution of an advanced architecture and transfer learning, a lightweight CNN baseline (four convolutional blocks with 32, 64, 128 and 256 channels, each followed by batch normalisation, ReLU and max pooling, with adaptive average pooling and a single linear output; 389,633 parameters) was trained from scratch with random Kaiming initialisation^43^ on the same data, splits and hyperparameters as the Inception-ResNet-V2 model.

#### Visualisation

To test the model on a real-life medical research question, the results of the above approach were analysed in a proof-of-principle setting in the context of clinical characteristics. The retrained Inception-ResNet-V2 model was used to evaluate heterogeneity at four hierarchical levels, including the lung function group and the patient, airway and patch levels (Fig. 2c). Two model outputs were used in the heterogeneity analyses: (1) the model’s learned features, which summarise relevant characteristics of airway image patches used for classification, and (2) probability predictions, which reflect the certainty of the model with respect to its prediction. For that, filtering was applied to ex-smokers ≥ 1 year for both COPD stage III and IV patients and controls to minimise variance due to smoking history.

#### Statistical analysis and visualisation of subgroup differences

At the highest hierarchical levels, including the lung function group and patient and airway levels, both distribution analysis and principal component analysis (PCA) were performed. Statistical testing is applied to analyse the degree of heterogeneity, although we acknowledge that our proof-of-principle study lacks a sufficiently powered dataset to make claims about significance. This statistical approach, therefore, cannot make strong claims regarding significance but rather demonstrates the potential value of downstream analyses derived from the image recognition outputs.

Instead of traditional testing, hierarchical bootstrapping^44,45^ is adopted for evaluating differences to mitigate the effects of two influential factors:

1. Nested data: The data have hierarchical structures, which violates the independence assumption between the data points of many statistical tests.
2. Class imbalance: The unequal ratio between the number of patches per lung compartment might lead to spurious results when different groups are compared if one compartment is overrepresented in one group compared with another.

In this hierarchical bootstrapping method, a total of 10,000 bootstrap samples are generated at the lung function level. For each bootstrap sample, five patients were randomly selected (with replacement) from both groups. Subsequently, 150 patches from each patient’s lung compartment were randomly chosen to evaluate differences. The differences in distribution, median and variance were assessed via the Mann‒Whitney U test, Mood’s test and Levene’s test, respectively, over all bootstrap repetitions. Second, PCA was applied to the model features to calculate and visualise the intercompartment distance among patients or airways. For this PCA application, the number of components was minimised based on a predefined minimum explained variance of 80%. The same significance tests were performed on the Euclidean distance between the submucosa and adventitia based on the principal components. Specifically, at the lung function group level, a mixed model on the model’s probabilities with a patient-level hierarchy variable and tissue compartment and COPD indicators as independent variables was estimated. All heterogeneity analyses were conducted using the training set, as it provides both the largest sample size and the most representative indication of the model’s learned patterns.

These heterogeneity analyses are exploratory in nature. Given the limited sample size of our proof-of-principle study, no multiple test correction was applied, and no definitive biological conclusions should be drawn from these results. Rather, these analyses demonstrate the methodology’s potential for future, adequately powered studies.

## Results

The results of each step of the developed image analysis methodology (Fig. 2) and the proof-of-principle application on COPD are discussed in sequential order.

### Data preprocessing

The preprocessing workflow used to prepare the WSIs for the image recognition model consisted of two key components: data acquisition and data cleaning (Fig. 2a, Supplementary Fig. 1). The data cleaning workflow was particularly important for controlling the areas from which the image recognition model learned. This study used a combination of three data cleaning steps that together aim to steer the model to learn mostly from amorphic tissue structures of the lung ECM: (1) nucleated and non-nucleated cell masking, such as lymphocytes and RBCs; (2) exclusion of noninformative images by a threshold on the minimum number of tissue pixels per patch; and (3) random imputation of the irrelevant areas (artefacts) by tissue-representative pixels.

Firstly, the effect of thresholding on the data quantity and quality was assessed by analysing the model performance for different thresholds (Supplementary Fig. 2). Increasing the sample size, which increased from 4.408 at the 80% threshold to over 45.451 at the 20% threshold, improved the performance. This effect levelled off for thresholds below 40%, as this led to images that were dominated by uninformative image areas, that is, with a large proportion of randomly imputed pixels. Similar validation AUCs (0.85) were obtained for the least stringent thresholds, in which images with at least 20% to 40% ECM tissue pixels were included. The performance decreased to an AUC of 0.77 at the 80% threshold, which was presumably due to a vastly decreasing training sample size. The least stringent 20% threshold was adopted, which provides the maximum sample size for downstream analysis. For this threshold, the resulting data distribution is shown in Supplementary Table 2.

**Fig. 3:**
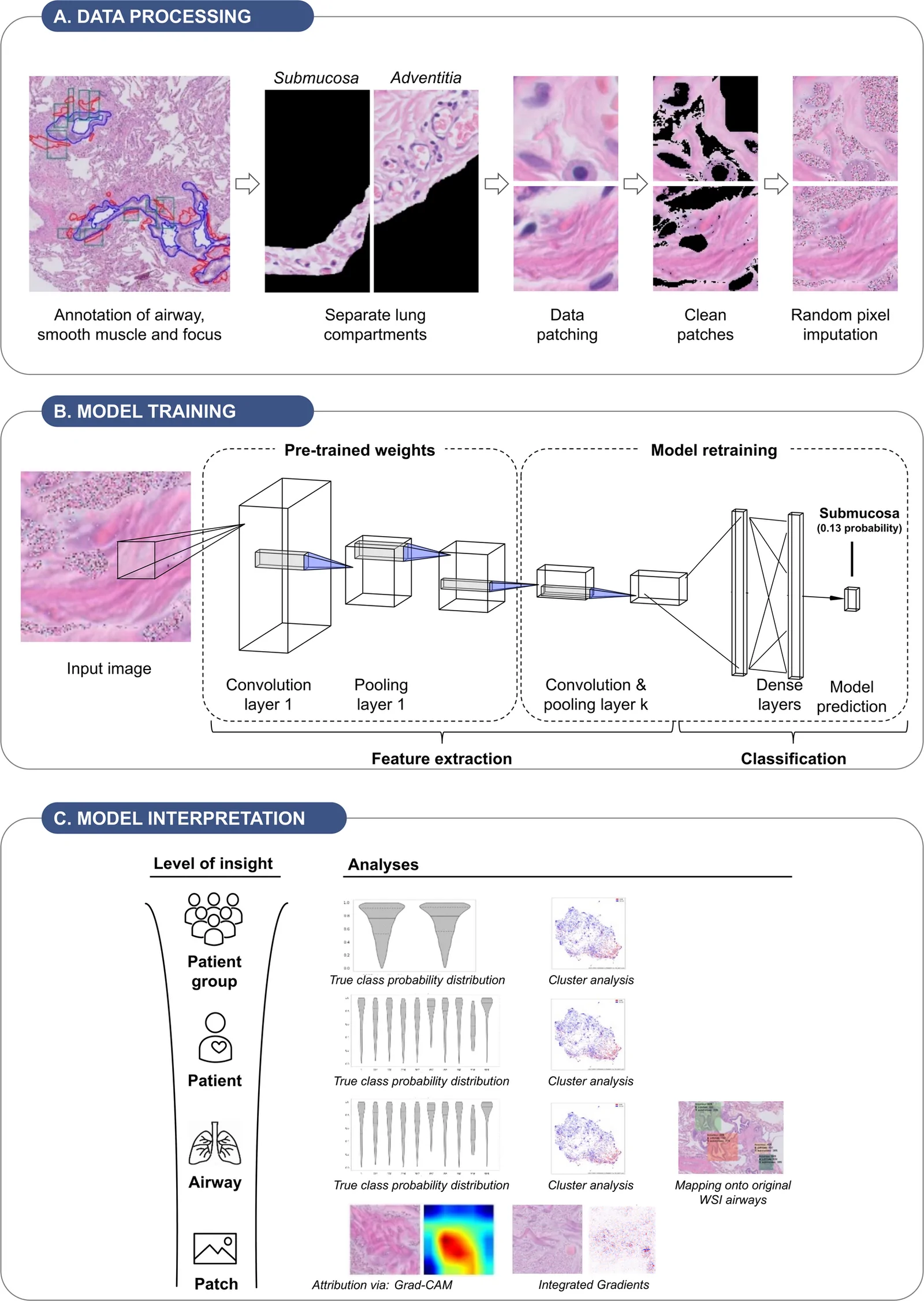
Illustrative comparison of limited and extensive image data preprocessing. An illustrative subset of image patches for a limited and extensive data preprocessing workflow from the submucosa and adventitia. In the limited data preprocessing workflow, there was no restriction on the percentage of black pixels, and no random pixel imputation was applied. Additionally, red blood cells (RBCs) are improperly cleaned via RGB ranges instead of via a segmentation algorithm. Displayed image patches were randomly selected from the top 1000 image patches with the highest true class probability score, which is the model output’s probability assigned to a patch’s observed or true class.

**Fig. 4:**
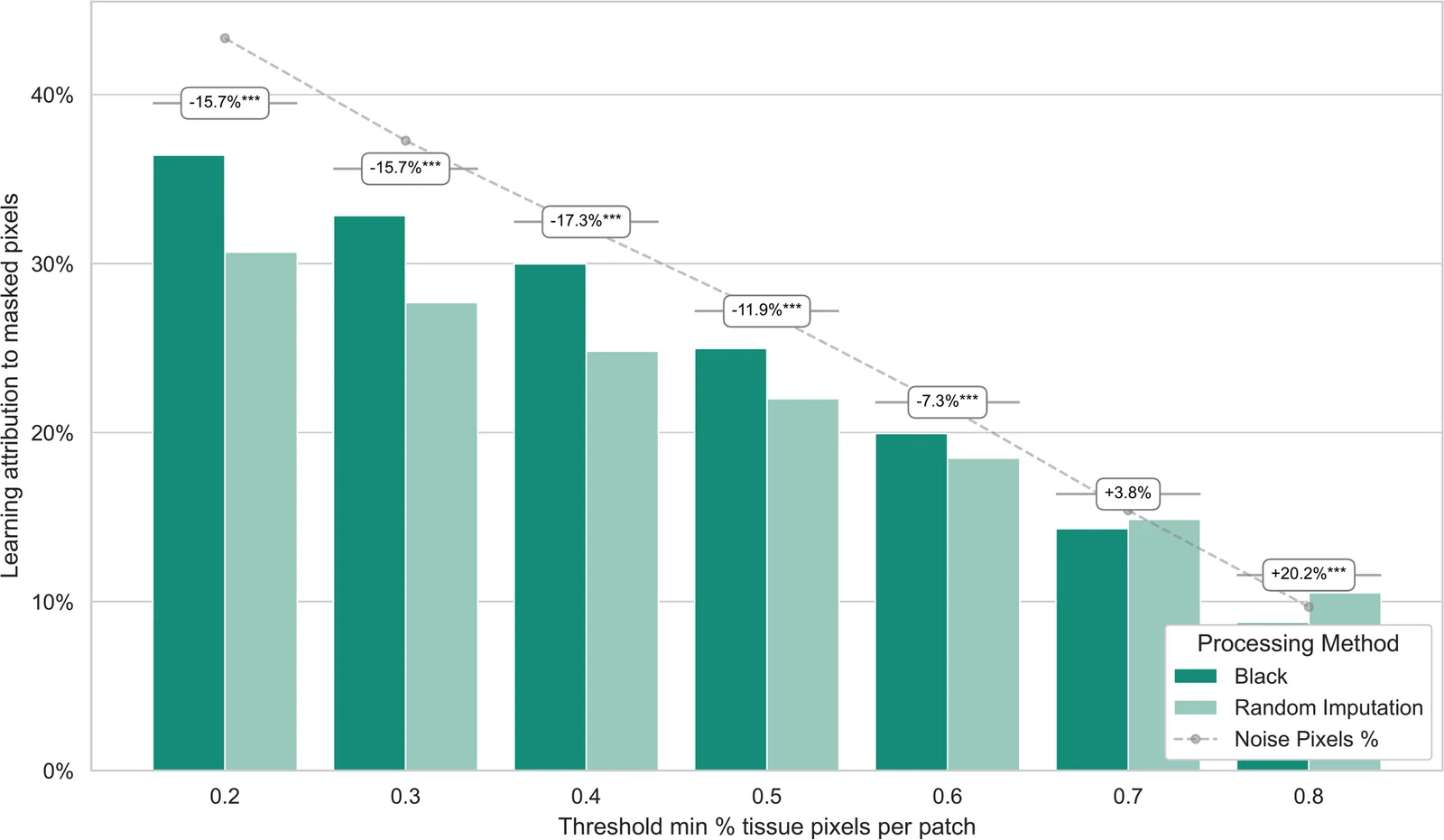
Comparison of model learning attribution to artefact regions under standard black versus randomised pixel preprocessing. This figure shows the percentage of learning attribution to artefact areas (masked pixels) as measured by Integrated Gradients. Lower values indicate that the model appropriately bases predictions less on artefact regions. A comparison between black and randomised artefact preprocessing is shown across varying percentages of irrelevant (masked) pixels via a model trained on images containing at least 20% tissue pixels. Statistical significance was assessed via a random effect mixed model with matched samples between the preprocessing method, accounting for data hierarchy through the subject and WSI. Significance: *p < 0.05, **p < 0.01, ***p < 0.001.

**Fig. 5:**
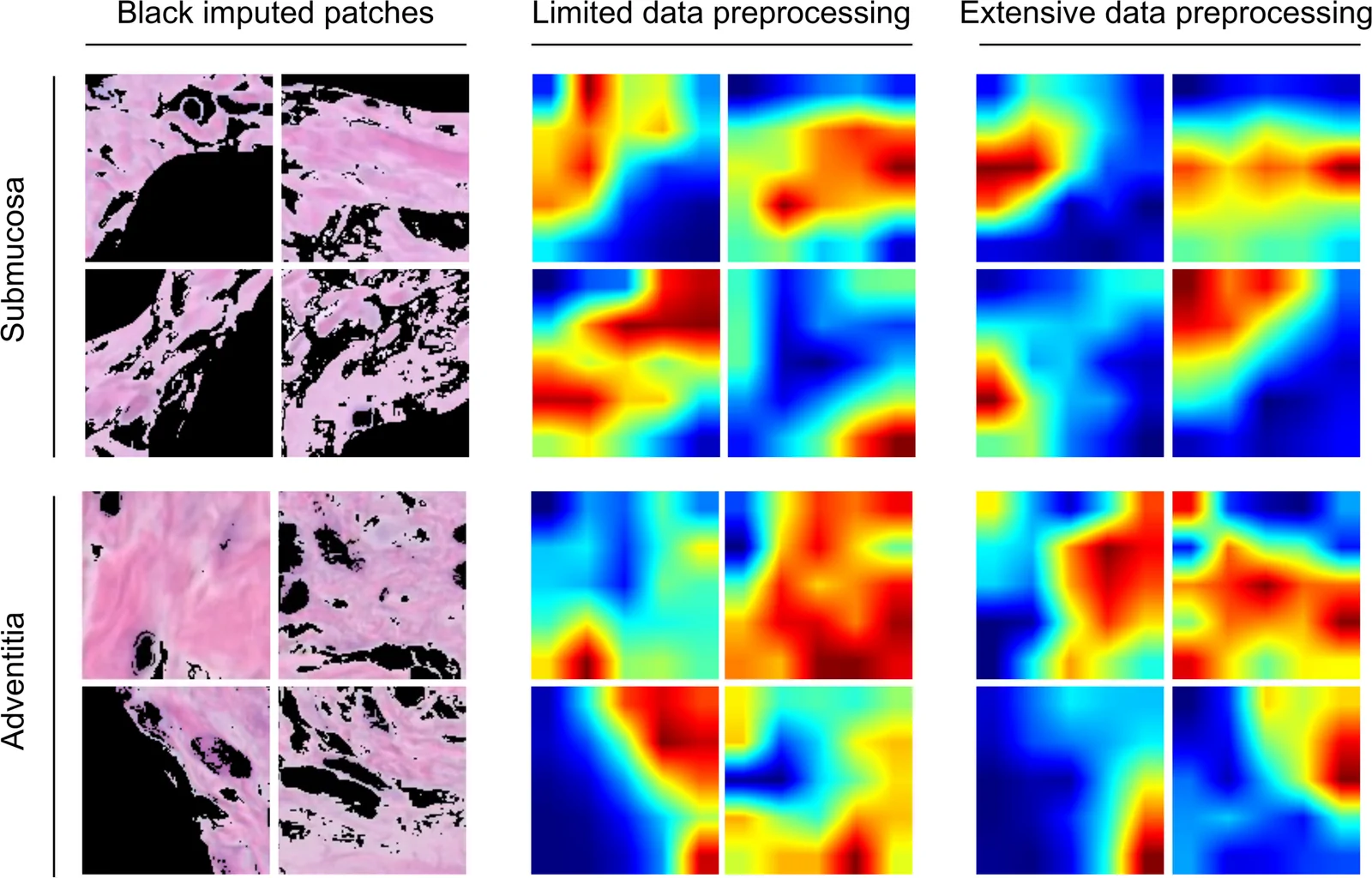
Grad-CAM attribution by two models with different data cleaning procedures. Two models with different data cleaning approaches (**a**) excluding random pixel imputation and (**b**) including random pixel imputation) were used to prepare the image patches. The importance of image areas for a model’s prediction is visualised using a colour scale from least important (blue) to most important (red). For example, in the top-right image, the red-coloured area in the middle indicates that the model bases its prediction mostly on this highlighted area, whereas the blue corners have the least influence. The patches are matched over the columns; that is, the original black imputed image on the top left links to the third and fifth feature discriminative plots on the first row.

Furthermore, the model’s learning behaviour is compared between a limited and an extensive (Supplementary Fig. 1) data cleaning workflow. In both cleaning workflows, nucleated and non-nucleated cells and other artefacts are removed; however, the limited workflow uses black imputation for those irrelevant areas, whereas the extensive workflow applies random tissue-representative pixel imputation. Figure 3 shows a visual representation of a subset of preprocessed submucosa and adventitia patches via both methods. Figure 4 shows the model’s learning patterns from masked pixels (artefacts) as measured by Integrated Gradients attribution, segmented by noise level, for the model trained on images with a minimum of 20% tissue pixel content. Random pixel imputation showed significantly better performance in directing the model to prioritise learning from tissue pixels rather than from artefact pixels for all thresholds up to 60%. This represents a desirable outcome of the training process. Similar patterns emerged when Grad-CAM was used for attribution calculations across all thresholds (Supplementary Fig. 10), although statistical significance was not achieved for two of the seven thresholds tested. Figure 5 depicts illustrative patches highlighting the Grad-CAM output, which indicates which patch areas are most important for model inference.

### Model training

After data preprocessing, the resulting data were used as input for the image recognition model. Different settings for model learning were evaluated, and the results are described below.

First, different transfer learning settings were evaluated to tune the pretrained Inception-ResNet-V2 model on the ImageNet database to the input data. Iterative increases in the number of retrained model layers were applied to optimise the model’s ability to learn patterns from image data. Table 1 shows the model performance for different retraining extents, varying from 3 to 781 retrained layers. A similar AUC of 0.85 on the validation set was obtained by retraining the Inception-ResNet-V2 model from the input layer onwards, such that the parameters of 781 layers were tuned to the dataset or from the A block onwards. Moreover, a comparable degree of overfitting (Table 1) was observed. Based on the similarity of the results, the model with 781 retrained layers was adopted for further analysis, as it yields more flexibility for further optimisation.

Based on the selected transfer learning strategy and data threshold, a sparse grid hyperparameter procedure was used for further optimisation (Supplementary Tables 7 and 8) to obtain the final model for downstream analysis. The training process and convergence of this model on both tissue compartments are shown in Supplementary Figs. 3 and 4. The recognition accuracies of the submucosa and adventitia were similar (validation accuracies of 76.89% and 76.63%, respectively). The performance development over time on the training and validation sets is shown in Supplementary Fig. 4.

In addition to different settings for model learning, the effect of the hierarchical data structure on the model’s generalisation performance was evaluated. Table 2 presents the results of strategies to split the dataset according to the three hierarchical levels, where multiple patches stem from the same WSI and multiple WSIs originate from the same patient. The best performance on the validation and test sets was observed when patches were randomly assigned to each set, indicating potential data leakage from patients occurring in different partitions. A lower performance was observed when dataset splits were informed by the data hierarchy, such that the datasets were independent at either the WSI or patient level, although the differences were modest. For all further analysis, patient-level data partitioning was adopted to reduce the influence of potential batch effects and leakage. The model was further optimised via a sparse grid, yielding a test AUC of 0.84, which was subsequently used for model interpretation and heterogeneity analysis.

### Heterogeneity analyses

The retrained Inception-ResNet-V2 model on tissue data enabled us to study heterogeneity in the lung airway ECM on multiple hierarchical levels, using two of the model outputs: (1) the model’s learned features, which summarised relevant characteristics of airway image patches, and (2) probability predictions, which reflect the model’s certainty about its prediction.

**Fig. 6:**
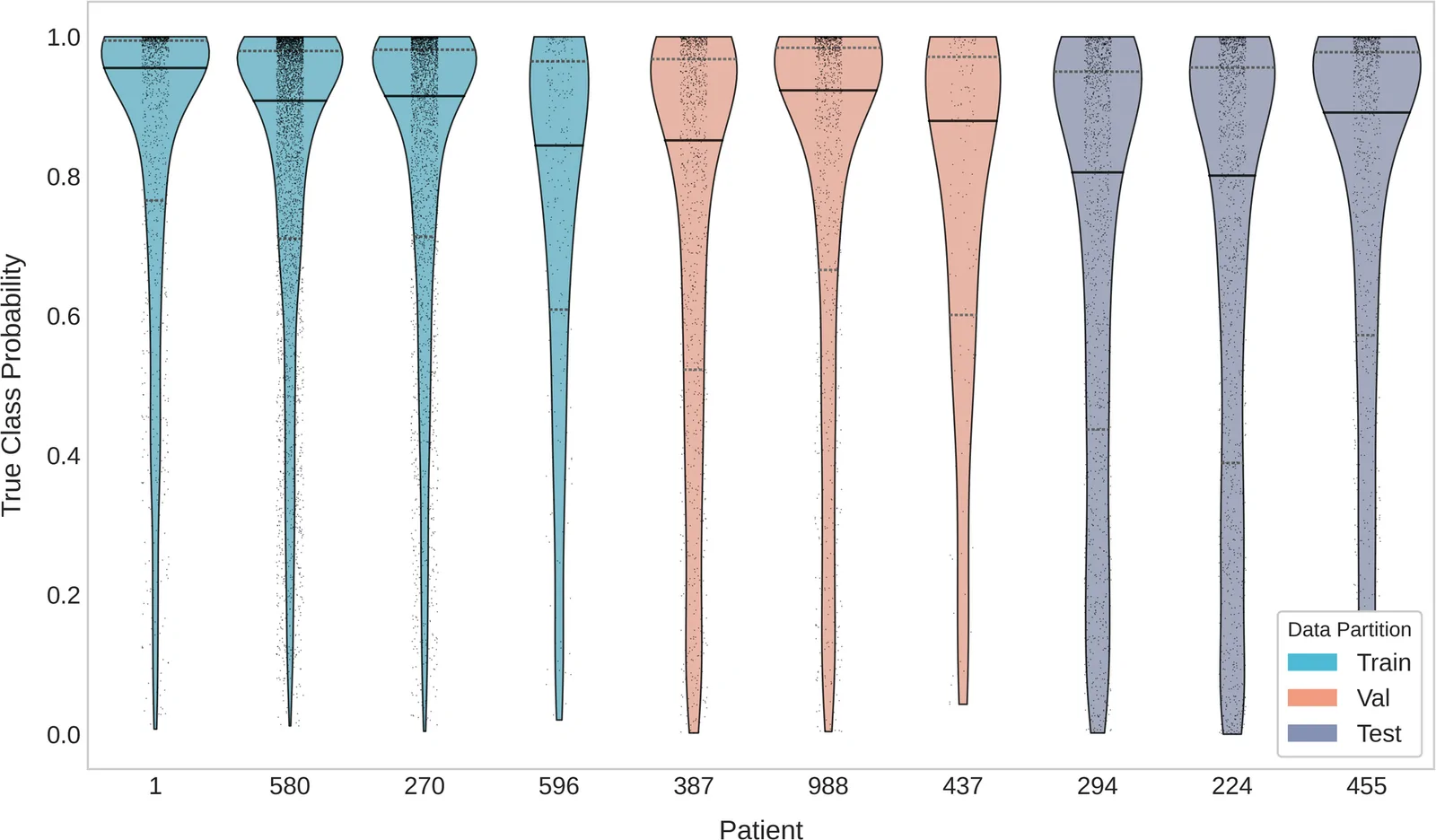
True class probability distribution aggregated at the patient level. All of the submucosa and adventitia patches of 1 patient with COPD stage III and 9 patients with COPD stage IV are shown. Probabilities closer to 1 indicate that the model’s prediction was close to the right tissue class, and vice versa. The solid horizontal lines in the distribution represent the medians, and the dashed lines represent the first and third quartiles (the 25th and 75th percentiles), respectively. In addition to disease status and being an ex-smoker ≥ 1 year, patients are also filtered for having at least 10 patches per compartment to draw a sensible solution. Patients from all the data partitions are shown.

At the patient level, heterogeneity could be observed and statistically assessed. For that, filtering was applied to ex-smokers ≥ 1 year to minimise variance due to smoking history. Figure 6 shows the differences in the probability distributions among the COPD patients. For example, for Patient 294, the true class probability is rather uniformly spread, whereas the distribution of Patient 455 is more skewed toward 1, implying that the model is more certain about its predictions for the latter patient. Accordingly, statistical tests revealed the observed distributional differences between the two patients at the 5% level. Similar analyses can be performed to obtain insights at the airway level, whereby the airway defines the grouping level to assess heterogeneity between airways.

As expected for a proof-of-principle setup, group analysis did not show significant differences, as for such goals, many more patients will have to be analysed to reach sufficient power. The present work proposes a methodological approach to analyse group heterogeneity and applies it to the data to demonstrate its use. No significant difference was observed between COPD stage III or IV patients and controls at a confidence level of 0.05, and the probability distributions between the two populations were comparable for both the submucosa and adventitia (Supplementary Figs. 5 and 6). In line with this observation, hierarchical bootstrapping could not reject the null hypothesis of an equal distribution, median and variance of the model’s probabilities between COPD patients and controls (Supplementary Table 3). Supplementary Table 3 also shows the impact of ignoring the hierarchical data structure, since the standard testing procedure incorrectly identifies distributional differences between COPD patients and control patients for the adventitia, contrary to the hierarchical bootstrapping approach. Similarly, the COPD coefficient of the mixed model on the model’s probabilities was not estimated to be significantly different from zero (Supplementary Table 4).

The absence of heterogeneity between COPD patients and controls was further confirmed by cluster analysis of the model’s learned features. Supplementary Fig. 7 displays the average lung compartment distance for the two patient groups based on the PCA analysis of the model’s learned features of the submucosa and adventitia. Although the distance between the two tissue compartments visually appeared to be greater for COPD patients than for controls, this difference was not statistically significant (Supplementary Table 5).

### Ablation analysis

Three preprocessing conditions were compared: no preprocessing (nonmasked original patches, test AUC 0.86; default hyperparameters), limited preprocessing (black masking without random imputation, test AUC 0.82; default hyperparameters), and the full pipeline (cell masking with random pixel imputation, test AUC 0.84; with hyperparameter optimisation). Note that the AUCs are not directly comparable across conditions because the first two were trained with default hyperparameters whilst the full pipeline was hyperparameter-optimised. Together with the attribution analysis (Figs. 4 and 5) and the noise resistance evaluation, these results indicate that the full preprocessing pipeline redirects model attention from artefact regions towards tissue pixels while preserving the discriminative ECM signal.

Three further ablation experiments are summarised in Supplementary Fig. 11. The shuffled-label negative control yielded a test AUC of 0.50 and a test loss of 0.693, confirming that the model’s performance of 0.84 on correctly labelled data reflects genuine label-associated tissue signal rather than artefacts introduced by the preprocessing pipeline. On the same patient-level split, a classical texture-based baseline (GLCM + LBP with linear SVM) achieved a test AUC of 0.71 and a lightweight CNN trained from scratch achieved 0.76, compared to 0.84 for the pretrained Inception-ResNet-V2. This stepwise hierarchy indicates that both the deep learning architecture and ImageNet-based transfer learning contribute meaningfully to the final performance, beyond what is achievable with handcrafted texture descriptors alone.

## Discussion

In this study, an innovative image analysis approach was developed to study histopathological WSIs. In contrast to most existing approaches that study cellular shapes, the aim was to analyse (pixel) patterns in non-cellular structures. The novelty of this work’s methodological framework is grounded in three main contributions. First, this study designed an extensive data processing pipeline with meticulous data cleaning procedures to focus solely on non-cellular structures. Second, a deep learning classification model was trained to distinguish two different connective tissue (ECM-containing) areas of human airways, i.e., the submucosa and adventitia, which was guided to emphasise learning from pixel-level patterns in the non-cellular tissue component rather than from artefacts or cellular structures. This was aided by transfer learning to leverage existing image recognition models and train them on new data. Finally, model visualisation techniques are used to interpret which information the image recognition model is based on. This study demonstrated the potential of this approach to explore novel research hypotheses by applying it in a proof-of-principle setup using COPD as a disease model. More generally, it confirms the critical importance of meticulous image preprocessing tailored to specific (biological) contexts and research questions.

AI analysis of amorphic structures has been underexplored, with most studies not actively aiming to focus purely on such structures. Extending existing approaches to such structures is, however, not trivial. In fact, many studies aim to develop automated diagnostic approaches instead of obtaining novel insights into biological structures. These approaches usually leverage all morphological data to obtain the most accurate diagnosis. The main value in such cases is that it is faster and more reproducible, and that it takes away more tedious parts of the pathologist’s work. Increasing insights into pathogenesis by extracting new information from a biological structure is less straightforward, especially when structural differences are not easily discernible, as in almost every standard H&E stain used. The first methods are emerging for studying the ECM via image recognition algorithms, such as TWOMBLI^46^, which quantifies global ECM patterns by generating a range of metrics. This method, however, is focused on global ECM metrics and does not enable analysis of specific tissue compartments, nor does its pipeline exclude unwanted artefacts that could introduce model bias. Previous studies have explored combining deep learning-generated features with handcrafted ones^47–50^. These approaches share our methodology of using neural networks to extract high-level abstract features to optimise predictive performance. However, the proposed method deliberately avoids assumptions about pre-known patterns and instead maximises learning directly from pixel patterns in WSI patches. A notable alternative approach uses graph neural networks to extract topological features^51^, contextualising information across patches. While this innovative method leverages deep learning capabilities, it contrasts with our focus on pattern differences rather than regional relationships.

The research concluded that an essential step in learning from histological WSIs is thorough data preprocessing. While deep learning models are generally regarded as hard to interpret, it is crucial to know and ensure that the model is not learning spurious or incorrect patterns. This also requires the involvement of field practitioners^52^, such as trained pathologists, to determine which image regions should be excluded from analysis. As deep learning models can collect all possible clues to make decisions, these models can still (unwantedly) learn from the presence of cell shapes such as RBCs, lymphocytes and the epithelium as well as from excluded (black-masked) areas. The risks associated with deceptive learning strategies in deep learning applications within histopathology are now beginning to receive warranted scrutiny^53^. Masking both nucleated and non-nucleated cells is important since the model-based inference on non-nucleated cells, such as clusters of RBCs, is performed when masking only nucleated cells. The application of the default model to nonmasked data yielded an improved test AUC of 0.86, compared with 0.84 for the optimised model with random imputation preprocessing. This increase in performance confirms that the model effectively incorporates masked regions during the learning phase. This study’s novel random-pixel imputation helped steer the model away from (unwantedly) learning from excluded areas resulting from splitting the airway compartments, but also from cleaned (black-masked) cellular shapes. Grad-CAM and Integrated Gradients visualisations indicate that this step forced the model to better learn from relevant image areas. This underscores the relevance of a proper data preprocessing pipeline.

Even with the certainty that the model is learning from the right areas within the images, deep learning models still require vast amounts of data to learn successfully. While data are abundant in domains such as radiology and pathology, labelled data with accurate area annotations are scarce and costly to acquire. Transfer learning is a popular and efficient way to overcome this issue, and its adoption within medical research is widespread^54,55^. Although retraining (also called fine-tuning) a transfer learning model is a common strategy within medicine^56,57^, studies infrequently evaluate different retraining strategies. Retraining more than only the last layer of a pretrained model, which is the dominant default strategy, can greatly improve model performance (Table 1). This is likely driven by the dissimilarity between the original ImageNet dataset on which Inception-ResNet-V2 is trained and the ECM images used in this study.

The combination of an extensive data preprocessing pipeline and a transfer learning strategy resulted in a model that was able to classify and separate the ECM in the submucosa and the adventitia accurately. This demonstrates the benefit of AI-powered image analyses, which are able to provide accurate predictions based on patterns in WSI patches, whereas such differences are difficult for the naked eye to discern. The immense resolution and depth of the information that WSIs offer facilitate this novel type of image analysis. Moreover, model interpretability is a known pitfall associated with deep learning techniques, which can hinder the adoption of such algorithms in practice. This study employed multiple model visualisation techniques to assess which image areas informed the model’s predictions. This methodology holds the potential for hypothesis-driven directions in future research.

The methodology was used in a proof-of-principle setup to demonstrate how the method can be used to analyse various research hypotheses and visualise group differences. This setup is easily scalable for other biological structures and disease models. Because our proof-of-principle study is underpowered, an adequately powered follow-up study would be required to test whether there are differences in ECM tissue structure in the airway compartments (i.e., airway submucosa and adventitia) in COPD patients compared with controls or to identify patient subgroups with characteristic ECM patterns. At the individual patient level, this work demonstrated the presence of heterogeneity, with substantial intraclass variation between patients in ECM patterns. In addition, the derived insights can be mapped back to the corresponding airway in the original WSI to connect the results to the broader lung context, as shown in Supplementary Fig. 8. However, as indicated above, to answer research questions regarding matrix changes in COPD patients properly, greater study power is needed. This methodology is readily adaptable to diverse pathological conditions and tissue types. The framework can be implemented by researchers across disciplines, requiring only appropriately annotated whole-slide images (WSIs) for application to their specific areas of interest.

Based on the findings of this study, researchers recommend the following to guide future research in addressing research questions in a robust and reproducible manner. Foremost, ensuring the availability of sample data is key for deep learning models to recognise and learn the patterns in image data^8^. Our study setup restricted our ability to detect subtle ECM pattern differences. This is further amplified by the heterogeneity in ECM pixel patterns. Future studies with larger cohorts would increase the statistical power, enabling improved assessment of ECM variations across disease stages.

The ablation experiments further support the validity of the methodology. The shuffled-label negative control confirmed that the model’s classification performance depends on genuine compartment-associated signal (AUC 0.50 vs. 0.84). A classical texture-based baseline (AUC 0.71) and a lightweight CNN trained from scratch without pretraining (AUC 0.76) provide a hierarchy of evidence: handcrafted texture features < CNN from scratch < pretrained Inception-ResNet-V2. This shows that both deep learning itself and ImageNet-based transfer learning contribute meaningfully to performance, despite the domain gap between natural images and histopathology. Together, these results provide converging evidence that the model learns from genuine tissue characteristics rather than from preprocessing artefacts or spurious correlations.

The control tissues in this study were obtained from surgical lung resections for lung cancer, using only regions distant from tumour involvement, all from ex-smokers, similar to the COPD-patients. While the COPD tissues originate from lung transplantation, providing relatively uniform samples, which were checked for any abnormalities by an experienced pulmonary pathologist (WT), nevertheless the control tissues may exhibit some baseline ECM remodelling associated with their clinical context. This potential confounding, together with the limited sample size, reinforces the proof-of-principle nature of the COPD–control comparison. Future studies applying this methodology should match or adjust for key covariates including age and sex, and associated pathology (type of tumour).

We acknowledge that the submucosa and adventitia are anatomically defined compartments that differ in tissue architecture and spatial context. Classification performance between these compartments does not, by itself, demonstrate that the model captures ECM-intrinsic biological differences. Anatomical context, tissue thickness, and spatial relationships may contribute to classification alongside ECM-intrinsic pixel patterns. The attribution analyses (Grad-CAM, IG) show that the preprocessed model focuses on tissue rather than artefact regions, but they cannot fully disentangle ECM-specific features from broader architectural cues.

In addition, three suggestions to further improve the methodological approach are offered: improving scalability, image staining combinations, and data cleaning. First, as manual annotation is highly time-consuming and often the limiting factor in sample size, the methodology should be complemented with automated segmentation algorithms to select areas of interest. This would enable rapid expansion of the methodology towards other diseases. There are promising steps taken in this direction, with recent advances in annotation-free deep learning approaches^58,59^, although these are currently focused only on diagnostic objectives and cellular structures. Second, the current study included only H&E-stained WSIs. In combination with other types of staining, immunohistochemistry aimed at identifying specific ECM proteins can be expected to provide more complete insights into disease heterogeneity^60–62^. Third, extending the data cleaning procedure could be of interest. Future work could explore more advanced imputation techniques, such as generative approaches, to further reduce artefact-related cues in the preprocessed patches. Finally, the use of more advanced segmentation tools may facilitate better masking of unwanted structures such as RBCs and thereby more reliable model learning.

## Conclusions

Taken together, the developed methodology provides a valuable addition to AI-empowered medical research. The methodology enables the exploration of new hypotheses and the sharpening of focus areas in an automated fashion. In that way, it can aid researchers or clinicians in new methods, complementing goal-oriented microscopic research and immunohistochemistry techniques. The preprocessing pipeline is reusable for applications involving histopathology data. In addition, the methodology provides another step towards a greater understanding of complex disease mechanisms, as insights into amorphic patterns could be connected to existing analyses of cellular shapes. With the ECM being an important yet often neglected component contributing to human disease pathogenesis, studying its different features helps to better understand complex biological dynamics of diseases, which could subsequently aid in diagnostic pathology. Finally, the approach is easily scalable for other tissue structures or disease models, for example, for analysing variations in the cancer-induced ECM, which could be related to clinical tumour-specific characteristics.

## Supplementary Information

Below is the link to the electronic supplementary material.

Supplementary Material 1

## Data Availability

The image patches, patient characteristics mapping, and an example QuPath annotation file are publicly available via Zenodo [doi:10.5281/zenodo.18267951]. These patches contain all image data necessary to replicate and extend the deep learning framework presented in this study. The original whole slide images (WSIs) are not publicly available as they were used for pathology diagnostics. All patient data were fully de-identified prior to analysis to ensure privacy. We acknowledge that without access to the original WSIs, certain upstream processing steps (including manual annotation boundaries, segmentation decisions, and patch extraction parameters) cannot be replicated from the published patches alone. The original WSIs are available upon reasonable request to the corresponding author to facilitate such replication.
