# Supplementary material for: A deep learning framework for histopathological analysis of pixel-level extracellular matrix variation in standard H&E-stained images
Source: Sci Rep. 2026 Jun 22;16:28453. doi: 10.1038/s41598-026-56138-9 (PMC13562512; doi:10.1038/s41598-026-56138-9)
Supplement: Supplementary file 1 [file 41598_2026_56138_MOESM1_ESM.docx]

# Supplementary figures

**Supplementary Figure 1. Detailed overview of the data preprocessing workflow**. Images were split, resulting in the extraction of focus areas in QuPath, (A) followed by data patching and cleaning in Python (B).

**Supplementary Figure 2. Data quantity per threshold.** In data preprocessing, a threshold is used on the minimum percentage of tissue pixels per image patch. For each threshold, the total data size and proportion of Submucosa patches after applying the threshold are depicted. The percentage of artefact pixels (masked) if 44% at the 20% threshold, while it drops to just 10% at the 80% threshold.

**Supplementary Figure 3. Training development of the Inception-ResNet-V2 model.** For model retraining starting from the input layer and optimised hyperparameters.

**Supplementary Figure 4. Accuracy development during training of the image classification model.** The Inception-ResNet-V2 model was run with 781 trainable layers on the training and validation set split per lung compartment: (A) Submucosa, (B) Adventitia.

**Supplementary Figure 5. True class probability distribution aggregated on the lung function level and split per lung compartment.** Split per tissue compartment: (A) Submucosa, (B) Adventitia. Figure includes patients from the training set only.

**Supplementary Figure 6. True class violin plot aggregated on lung function level and split per lung compartment.** Split per tissue compartment: (A) Submucosa, (B) Adventitia. Figure includes patients from the training set only.

 **Supplementary Figure 7. Principal components calculated on the model’s features averaged per patient and lung compartment, split per lung function.** (A) COPD stage III or IV patients (B) controls. Dot labels denote (anonymised) patient numbers to connect the principal components of both lung compartments corresponding to the same patient. The closer the PCs of the two lung tissues are for a patient, the higher the similarity in the ECM patterns of both tissues. Beyond disease status, patients are also filtered for having at least 10 patches per compartment to draw a sensible solution.

**Supplementary Figure 8. Model performance mapped to the original WSI on the airway level.** Illustration of how the accuracy of patch predictions can be mapped back to the airways in the original WSIs, which could be used for broader tissue analysis. Colours (red - low to green high) indicate how well the model distinguishes submucosa from adventitia. Poorer performance could suggest deviations in the ECM patterns of the corresponding airway.

 **Supplementary Figure 9. Inception-ResNet-V2 architecture.** The Inception-ResNet-V2 network forms the backbone of this image recognition application, to distinguish the lung compartments Submucosa and Adventitia. Two types of transfer learning are tested to tune this network to the lung tissue data: 1) More than just the final classification layer is retrained. The number of retrained layers can be selected and optimised. 2) Only the final classification layer is retrained; all other layers’ parameters are frozen. The blocks A-C refer to the architectural blocks that form the Inception-ResNet-V2 model. Block A is designed for capturing fine-grained, low-level features, whereas Blocks B and C capture more medium and high-level and abstract features, respectively. Image is adjusted from Google’s blog on this architecture^1^.

**Supplementary Figure 10. Grad-CAM attribution from artefact pixels for a standard black versus randomised** **pixel preprocessing.** This figure shows the percentage of learning attribution to artefact areas (masked pixels) as measured by Grad-CAM. Lower values indicate the model appropriately bases predictions less on artefact regions. Statistical significance was assessed using a random effect mixed model with matched samples between preprocessing methods, accounting for data hierarchy through subject and WSI. Significance: *p<0.05, **p<0.01, ***p<0.001

 **Supplementary Figure 11. Hierarchy of test AUC across the ablation models.** Test AUC of four models trained on the same patient-level partition and identical preprocessing pipeline (random pixel imputation, 20% tissue threshold; 26,152 / 8,752 / 9,919 patches in train / validation / test): shuffled-label negative control (0.50), classical texture baseline (GLCM + LBP + linear SVM, 0.71), lightweight CNN trained from scratch (~0.39 M parameters, Kaiming initialisation, 0.76), and pretrained Inception-ResNet-V2 with 781 retrained layers (0.84). Each step in the hierarchy contributes meaningfully: genuine compartment signal exists, deep learning extracts more than handcrafted features, and ImageNet pretraining adds further improvement.

# Supplementary tables

|  |  | **Intersection on lung function** | | |
| --- | --- | --- | --- | --- |
|  | **Total** | GOLD stage III/IV | Gold stage I/II or low FEV1 | Control |
| **WSIs (n)** | 162 | 35 | 36 | 91 |
| **Patients (n)** | 75 | 10 | 18 | 47 |
| **Gender male** | 82 (51%) | 14 (40%) | 16 (44%) | 52 (57%) |
| **Age (years)** | 59.9 (11.93) | 58.1 (7.40) | 61.8 (11.43) | 59.9 (13.45) |
| **Smoking history** |  |  |  |  |
| Current smokers | 43 (26.5%) | - | 14 (8.6%) | 29 (17.9%) |
| Ex-smokers < 1 year | 8 (4.9%) | - | 1 (0.62%) | 7 (4.3%) |
| Ex-smokers ≥ 1 year | 75 (46.3%) | 35 (21.6%) | 13 (8.0%) | 27 (16.7%) |
| Never smokers | 35 (21.6%) | - | 8 (4.9%) | 27 (16.7%) |
| Unknown | 1 (0.6%) | - | - | 1 (0.6%) |

**Supplementary Table 1. Descriptive statistics on WSIs and patient characteristics.** Continuous variables are presented as mean (SD), and categorical variables are presented as frequency (percentage of total). The control group includes patients diagnosed with borderline or without COPD diagnosis and satisfying lung function criteria FEV1 % prediction (forced expiratory volume in 1 second compared to predicted) ≥ 68, and FEV1/FVC prebronchodilator (ratio of the forced expiratory volume in the first second to the forced vital capacity of the lungs) ≥ 68. Gold stage I/II or low FEV1 includes patients diagnosed with COPD stage I/II or FEV1 % prediction < 68 or FEV1/FVC ratio prebronchodilator < 68.

|  | **Lung function** | | |  |
| --- | --- | --- | --- | --- |
|  | GOLD stage III/IV | Gold stage I/II or low FEV1 | Control | **Total** |
| **Smoking history** |  |  |  |  |
| Current smokers | - | 4.2k (9%) | 8.6k (19%) | 12.8k (28%) |
| Ex-smokers < 1 year | - | 0.1k (0%) | 1.2k (3%) | 1.3k (3%) |
| Ex-smokers ≥ 1 year | 12.8k (28%) | 4.5k (10%) | 7.8k (17%) | 25.1k (55%) |
| Never smokers | - | 1.4k (3%) | 4.6k (10%) | 6.0k (13%) |
| Unknown | - | - | 0.3k (1%) | 0.3k (1%) |
| **Total** | 12.8k (28%) | 10.1k (22%) | 22.5k (49%) | 45.4k (100%) |

**Supplementary Table 2. Data distribution split on patient characteristics for a data cleaning threshold of a minimum 20% tissue pixels per patch.** Quantity and percentage of patches available for model training, validation and interpretation, after applying the preprocessing workflow.

| **Statistical test** | **Testing** | **P-value standard testing** | **P-value hierarchical bootstrapping** |
| --- | --- | --- | --- |
| Submucosa |  |  |  |
| Mann-Whitney U-test | Distribution | 3.37e-15 | 0.2653 |
| Mood’s test | Median | 1.01e-11 | 0.3800 |
| Levene’s test | Variance | 2.24e-2 | 0.4929 |
| Adventitia |  |  |  |
| Mann-Whitney U-test | Distribution | 7.19e-14 | 0.2051 |
| Mood’s test | Median | 2.87e-08 | 0.2724 |
| Levene’s test | Variance | 8.08e-05 | 0.3734 |

**Supplementary Table 3. Significance test results for different true class probability distributions.** Comparing population groups, COPD stage III and IV patients versus controls, filtered on ex-smokers ≥ 1 year. P-value with hierarchical bootstrapping takes the hierarchical and non-independent nature of the image patches into account. Results based on the training set for increased power, yet similar results were observed for the validation set.

| **Variable** | **Coefficient** | **Standard error** | **Z**  **(P > \|z\|)** | **Confidence interval**  **[0.025 – 0.975]** |
| --- | --- | --- | --- | --- |
| Intercept | 0.793 | 0.018 | 44.395  (0.000) | [0.758 – 0.828] |
| COPD indicator | 0.042 | 0.022 | 1.853 (0.064) | [-0.002 – 0.086] |
| Tissue compartment | 0.013 | 0.005 | 2.762  (0.006) | [0.004 – 0.022] |
| Patient hierarchy | 0.001 | 0.003 |  |  |

**Supplementary Table 4. Multi-level mixed effects model results on the Inception-ResNet-V2 output probabilities**. Including a patient-level hierarchy variable, tissue compartment and COPD indicator as independent variables. The group sizes refer to the number of patches available for patients in the hierarchy, filtered on ex-smokers ≥ 1 year in the training set. Total number of observations (image patches) was 13107, divided over 15 patients in the mixed model, with the number of patches per patient varying between 157 and 3548 and a mean number of patches of 874.

| **Statistical test** | **Testing** | **P-value** |
| --- | --- | --- |
| Mann-Whitney U-test | Distribution | 0.164 |
| Mood’s test | Median | 0.689 |
| Levene’s test | Variance | 0.335 |

**Supplementary Table 5. Significance test results on the feature distance between lung compartments.** Quantifying the lung compartment distance using the model’s learned features of the submucosa and adventitia, for COPD stage III or IV patients compared to controls, filtered on ex-smokers ≥ 1 year. The feature distances were averaged on the patient level, having at least 10 patches per compartment, and only including patients from the training set. In total, four patients with COPD stage III or IV with a total of 3.1k and 4.2k submucosa and adventitia patches, respectively, and seven controls with total of 1.4k and 2.1k submucosa and adventitia patches respectively were included. Similar results were observed when including all patients across data partitions.

| **Non-ECM structure** | **Main color** | **Red range** | **Green range** | **Blue range** |
| --- | --- | --- | --- | --- |
| Epithelium and lymphocytes | Purple | 50 – 189 | 32 – 212 | 90 - 220 |
|  |  | Additional condition: blue – red ≥ 10 | | |
| Carbon particles | Black | 48 – 124 | 30 – 102 | 42 – 103 |
| Background | Light grey | 212 – 238 | 200 – 234 | 224 - 250 |

**Supplementary Table 6. RGB ranges used for masking non-ECM structures.** RGB ranges have been used to mask artefacts like epithelium, lymphocytes, carbon particles and background in addition to RBC cleaning via a classification algorithm.

| **Hyperparameter** | **Configuration** |
| --- | --- |
| Loss function | Binary cross-entropy |
| Learning rate | 0.0005 |
| Reduce learning rate on plateau | Median |
| Patience | 10 |
| Cooldown | 0 |
| Reduction factor | 0.1 |
| Minimum delta | 0.0001 |
| Minimum learning rate | 0.00001 |
| Adam optimiser |  |
| Beta – first decay parameter | 0.9 |
| Beta – second decay parameter | 0.999 |
| Dropout rate | 0 |
| Epochs | 100 |
| Batch size | 128 |

**Supplementary Table 7. Optimised hyperparameters used to train the Inception-ResNet-V2 models.** Hyperparameters have been optimised via a sparse grid, see also Supplementary Table 8.

| **Step** | **Hyperparameter** | **Grid configuration** |
| --- | --- | --- |
| 1 | Learning rate | 0.005, 0.001, **0.0005**, 0.0001 |
|  | Batch size | 32, 64, **128** |
| 2 | Data augmentation type | None, **extensive ^A^** |
|  | Dropout rate | **0**, 0.2, 0.4, 0.6, 0.8 |

**Supplementary Table 8. Multi-step sparse grid for hyperparameter optimisation.** To reduce computational load, hyperparameters were optimised in two steps. First, via a grid with the most important hyperparameters for a model such as Inception-ResNet-V2, e.g. learning rate and batch size. The optimal values (bold in the table) were selected based on the validation AUC and fixed for Step 2, which then subsequently optimised the other values. ^A^ Included the following steps: random rotations up to 30 degrees, horizontal and vertical flips, random cropping with resizing (maintaining 70-100% of original size), and colour adjustments to brightness, contrast, saturation, and hue. These augmentations are applied only to the training data.

| **Partition split** | **Train AUC** | **Validation AUC** | **Test AUC** |
| --- | --- | --- | --- |
| Split 1 (same as Table 1) | 0.9369 | 0.8472 | 0.8215 |
| Split 2 | 0.9020 | 0.8494 | 0.8148 |
| Split 3 | 0.9000 | 0.8546 | 0.8314 |

**Supplementary Table 9.** **Sensitivity of patient-level splits in model performance.** Splits are achieved by adjusting the random seed of the splitting algorithm. Given the relatively small number of patients, stratification of the COPD group is applied in making the splits to ensure that all partitions have a similar percentage of COPD patients. All results were based on the Inception-ResNet-V2 with 781 retrainable layers and optimised hyperparameters in Supplementary Table 6.

# Supplementary References

1.	Alemi A. Improving Inception and Image Classification in TensorFlow. Google Research. 2016. Accessed November 3, 2024. https://research.google/blog/improving-inception-and-image-classification-in-tensorflow/
